# Lessons From a Quality Improvement Project Aiming to Reduce Missed Outpatient Appointments in a Community Mental Health Regional Centre

**DOI:** 10.1192/bjo.2026.11377

**Published:** 2026-06-30

**Authors:** Nia Fenn, Terrenjit Gill, Hami Zaman, Jagdev Thukral

**Affiliations:** 1 King's College London, London, United Kingdom; 2 Oxleas NHS Foundation Trust, London, United Kingdom

## Abstract

**Aims:**

High “Did Not Attend” (DNA) rates in mental health care lead to poorer patient outcomes and significant financial costs. In Community Mental Health Teams (CMHTs), high DNA rates are not uncommon due to the complexity of patient needs and operational pressures. Within the Anxiety, Depression, Affective, Personality Disorders and Trauma (ADAPT) service in Bromley East, a baseline DNA rate of approximately 25% represented a significant clinical and operational challenge. This project aims to reduce DNA rates from 25% to 20% within 4 months (Oct 2024–Feb 2025).

**Methods:**

A local service evaluation was initially conducted using a multi-modal approach, including staff interviews, data analysis in collaboration with the administration team, andpatient questionnaires. Three PDSA cycles were subsequently implemented. The first two focused on improving staff understanding of DNA documentation. Intervention 1 involved an email and discussion in the multidisciplinary team meeting, outlining accurate DNA reporting and important considerations. Intervention 2 included posters in staff offices for reinforcement. Intervention 3 targeted patient behaviour with posters in the reception area and leaflets distributed. Weekly DNA rate percentages were retrospectively collected.

**Results:**

The interventions did not result in a sustained decrease in DNA rates (median=22.7%). The run chart indicated common-cause variation, suggesting that week-on-week changes were due to random variation.

**Conclusion:**

No sustained or significant decrease in DNA rates was observed. Incorrect documentation was likely overestimated as a contributing factor. The limited impact of low-intensity interventions highlights the multifactorial nature of attendance behaviour within CMHT populations, including operational constraints, difficulties in contacting first-appointment non-attendees, and broader bio-psycho-social factors.

Future work should prioritise early stakeholder involvement and targeted, engagement-focused strategies rather than infrastructure-focused reminders alone. While digital tools may offer additional support, they are unlikely to be effective without parallel investment in personalised, relational, and system-level change. Importantly, improving attendance metrics alone does not necessarily equate to improved care.

Although the project did not achieve its initial target, it increased staff awareness, prompted multidisciplinary discussion, and provided valuable learning in quality improvement methodology. Reporting negative or neutral findings is vital to avoid repeating ineffective strategies and to inform the development of more effective future interventions.

